# Covalent Organic Framework with 3D Ordered Channel and Multi-Functional Groups Endows Zn Anode with Superior Stability

**DOI:** 10.1007/s40820-023-01278-0

**Published:** 2024-01-04

**Authors:** Bin Li, Pengchao Ruan, Xieyu Xu, Zhangxing He, Xinyan Zhu, Liang Pan, Ziyu Peng, Yangyang Liu, Peng Zhou, Bingan Lu, Lei Dai, Jiang Zhou

**Affiliations:** 1 School of Chemical Engineering, North China University of Science and Technology, Tangshan, 063009 People’s Republic of China; 2 School of Materials Science and Engineering, Central South University, Changsha, 410083 People’s Republic of China; 3 State Key Laboratory for Mechanical Behavior of Materials, Xi’an Jiaotong University, Xi’an, 710049 People’s Republic of China; 4 Hunan Provincial Key Defense Laboratory of High Temperature Wear-Resisting Materials and Preparation Technology, Hunan University of Science and Technology, Xiangtan, 411201 People’s Republic of China; 5 School of Physics and Electronics, Hunan University, Changsha, 410082 People’s Republic of China

**Keywords:** Aqueous Zn ion batteries, Covalent organic framework, Interfacial modification, Zn ion flux regulation, Desolvation effect

## Abstract

Achieving a highly robust zinc (Zn) metal anode is extremely important for improving the performance of aqueous Zn-ion batteries (AZIBs) for advancing “carbon neutrality” society, which is hampered by the uncontrollable growth of Zn dendrite and severe side reactions including hydrogen evolution reaction, corrosion, and passivation, etc. Herein, an interlayer containing fluorinated zincophilic covalent organic framework with sulfonic acid groups (COF-S-F) is developed on Zn metal (Zn@COF-S-F) as the artificial solid electrolyte interface (SEI). Sulfonic acid group (− SO_3_H) in COF-S-F can effectively ameliorate the desolvation process of hydrated Zn ions, and the three-dimensional channel with fluoride group (-F) can provide interconnected channels for the favorable transport of Zn ions with ion-confinement effects, endowing Zn@COF-S-F with dendrite-free morphology and suppressed side reactions. Consequently, Zn@COF-S-F symmetric cell can stably cycle for 1,000 h with low average hysteresis voltage (50.5 mV) at the current density of 1.5 mA cm^−2^. Zn@COF-S-F|MnO_2_ cell delivers the discharge specific capacity of 206.8 mAh g^−1^ at the current density of 1.2 A g^−1^ after 800 cycles with high-capacity retention (87.9%). Enlightening, building artificial SEI on metallic Zn surface with targeted design has been proved as the effective strategy to foster the practical application of high-performance AZIBs.

**Supplementary Information:**

The online version contains supplementary material available at 10.1007/s40820-023-01278-0.

## Highlights

- A fluorinated zincophilic covalent organic framework (COF-S-F) with sulfonic acid group (-SO_3_H) is prepared on the surface of Zn anode, which promotes the desolvation of hydrated Zn ions and inhibits the side reactions.
- The highly electronegative -F group in COF-S-F promotes fast and uniform transport of Zn ions along the interconnected channels, which contributes to the uniform electrodeposition process of Zn metal.
- Zn@COF-S-F symmetric cell achieves a superior stability of 1,000 h and Zn@COF-S-F|MnO_2_ cell delivers high specific capacity of 206.8 mAh g^−1^ at current density of 1.2 A g^−1^.

**Supplementary Information:**

The online version contains supplementary material available at 10.1007/s40820-023-01278-0.

## Graphical abstract

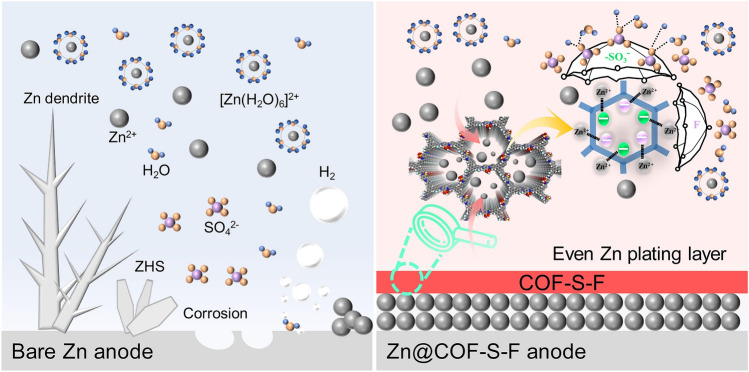

## Introduction

Along with the advancement of a “carbon neutrality” society, renewable energy represented by wind energy, solar energy, and tidal energy has been regarded as the alternative energy source for the growing energy demand and environmental degradation. However, its extensive application is still fatally challenged by the intermittent and spatiotemporally unstable characteristics, which is urgently needed to develop a highly reliable energy storage system [1–3]. Rechargeable lithium-ion batteries are widely used in electronic products and take in the major energy market due to their high energy density [4], but the limited abundance of Li resources and toxic electrolytes restrict their further development [5]. Aqueous Zn-ion batteries (AZIBs) featuring low-cost, low anodic redox potential, and high anodic theoretical capacity come into being [6–9], which are considered suitable large-scale energy storage applications to make full use of renewable energy [10–13]. However, the electrochemical performance of AZIBs is not as expected, which is attributed to the growth of Zn dendrites due to uneven distribution of the electric field and the notorious “tip effect,” as well as the corrosion and passivation of Zn anode from the hydrogen evolution reaction (HER) [14–17].

Very recently, massive researches have been ongoing to overcome these challenges and optimize the practical application of AZIBs, which can be sorted into constructing artificial solid electrolyte interface (SEI), Zn anode structural design, Zn alloy, and electrolyte optimization, etc. [18–26]. Among them, building artificial SEI on Zn metal surface has been considered as the most effective strategy to uniform the electrodeposition of Zn metal through the regulation of interfacial electric field as well as the inhibition of the side reactions. For instance, the barium titanate SEI with porous structure can regulate the diffusion of Zn ions and thus uniform the electrodeposition, while the further development is hampered by its low strength and brittleness [27]. Hence, flexibility with higher strength is needed for the artificial SEI to adapt the interface-morphological evolution during the electrodeposition process of Zn anode. Meanwhile, the abundant polar bonds (-CN, etc.) in commercial solvent-free cyanoacrylate SEI greatly inhibit the occurrence of side reactions [28]. Apart from these, covalent organic frameworks (COFs) with the advantages of porosity, semi-conductivity, and adjustable chemical properties, have been promoted as promising materials for building artificial SEI on Zn metal to jointly tackle the complicated interfacial issues [29, 30]. Notably, three-dimensional (3D) COFs can provide interconnected channels for the favorable transport of Zn ions across the artificial SEI. Therefore, the development of multi-functional COFs as coating using molecular engineering has great potential in the construction of Zn anode for high-performance AZIBs.

Herein, we propose a fluorinated zincophilic COF with sulfonic acid groups (-SO_3_H) and -F group as SEI for Zn anode (Zn@COF-S-F), which can modulate the desolvation behavior of Zn ions as well as the electrodeposition of Zn metal. In detail, the abundant negative-charged polar -SO_3_H groups in COF layer can facilitate the desolvation process of Zn ions that coordinate with water molecules (H_2_O). Moreover, COF with 3D porous structure can modulate the flux of Zn ions through the ion-confinement effect to achieve a uniform and stable electrodeposition of Zn. The strong interaction between -F group and H_2_O can effectively prevent the permeation of electrolytes and thus improve the corrosion resistance of Zn anode. Consequently, Zn@COF-S-F symmetric cell exhibits a cycle life of 1,000 h at the current density of 1.5 mA cm^−2^ with an average hysteresis voltage of 50.5 mV. Zn@COF-S-F|MnO_2_ cell can deliver a high specific capacity of 206.8 mAh g^−1^ at the current density of 1.2 A g^−1^, which can maintain 87.9% of capacity after 800 cycles.

## Experimental

### Materials

1,3,5-Tris(3-fluoro-4-formylphenyl) benzene was purchased from Zhengzhou Alpha Chemical Co., Ltd. 2,5-Diaminobenzenesulfonic acid and o-dichlorobenzen were purchased from Shanghai Aladdin Biochemical Technology Co., Ltd. Other chemical substances were of analytical grade and had not undergone other treatments.

### Preparation of Materials

#### Pretreatment of COF-S-F

26.67 mg of 1,3,5-tris(3-fluoro-4-formylphenyl) benzene was dissolved with 16.94 mg of 2,5-diaminobenzenesulfonic acid in a mixture of 15 mL of o-dichlorobenzene and 15 mL of n-butanol, followed by sonication for 30 min to disperse the mixture. After sonication, the sonication was repeated for 10 min with 0.3 mL of 6 M acetic acid and then transferred to an autoclave and heated at 120 °C for 72 h. The resulting sample was cooled naturally, soaked in tetrahydrofuran for 12 h and subsequently centrifuged in ethanol and rinsed several times with deionized water. Finally, the precipitate was dried in an oven at 80 °C to obtain F-SO_3_H-COF powder, denoted as COF-S-F.

#### Preparation of COF-S-F-Coated Zn Foil (Zn@COF-S-F)

The commercial Zn foil was wiped using alcohol, dried and prepared for use. The obtained COF-S-F and PVDF were mixed according to a mass ratio of 9:1, the content of PVDF is extremely low, only 10% of the total mass, as a way to reduce the impact of the F element in PVDF on the cells. Then an appropriate amount of N-methylpyrrolidone was dropped into the above mixture to form a slurry, which was applied to the surface of the pristine Zn foil using a four-sided preparator and dried naturally for 12 h to obtain a Zn foil coated by COF-S-F, which was cut into 14-mm-diameter disks to serve as Zn anode, noted as Zn@COF-S-F. Herein, the mass loading of COF-S-F coating was 1.2 ~ 1.5 mg.

#### ***Synthesis of MnO***_***2***_*** Cathode Material***

0.768 g of MnSO_4_·H_2_O and 0.476 g of KMnO_4_ were dissolved in 15 mL of distilled water and each stirred at room temperature for 15 min. The resulting KMnO_4_ solution was then added dropwise to the above MnSO_4_ solution and stirred again for 30 min. The mixture was transferred to an autoclave with a Teflon liner and heated at 160 °C for 12 h. After natural cooling, the resulting precipitate was centrifuged and washed repeatedly with deionized water. Finally, the precipitate was dried in an oven at 80 °C to obtain MnO_2_. The obtained MnO_2_, Super P, and PVDF were mixed in a 7:2:1 mass ratio. An appropriate amount of NMP was dropped into the above mixture to form a slurry, which was then applied to a disk-shaped stainless-steel mesh and dried in an oven at 80 °C. After weighing on an electronic balance, a MnO_2_ loading mass of about 1.2 ~ 1.6 mg was obtained.

### Characterizations

X-ray diffraction (XRD) was used to investigate the crystalline phase of samples by a D8 Advance A25 Instrument (Bruker, Germany). X-ray photoelectron spectroscopy (XPS) analysis of samples was conducted using k-Alpha Plus (Thermo Fisher, USA). Fourier transform infrared spectrometer (FTIR) (TENSOR II, Bruker, Germany) was used to characterize the chemical structure of the introduced polymer layer. Scanning electron microscopy (SEM, JSM-IT100, JEOL, Japan) was used to examine the morphology of samples. N_2_ adsorption and desorption isotherms and Brurauer-Emmerr-Teller (BET) surface areas were collected by a specific surface area analyzer (3H-2000PM1, Beijing, China). A contact angle goniometer (JC2001, Shanghai) was carried out on a graphite plate covering the samples to study the hydrophilicity.

### Electrochemical Measurements

The electrochemical performances of Zn|MnO_2_ full cell, Zn|Cu asymmetric cell, and Zn//Zn symmetric cell were evaluated by using a CR2016 coin-shaped cell. All cells were assembled in the air at room temperature. Zn|MnO_2_ full cell was assembled with bare Zn or Zn@COF-S-F as the anode, α-MnO_2_ electrode as the cathode, 2 M ZnSO_4_ + 0.1 M MnSO_4_ solution as the electrolyte, and the glass fiber (φ = 18 mm) as the separator. The symmetric cell was assembled with Zn foil (bare Zn or Zn@COF-S-F) and 2 M ZnSO_4_ electrolyte. The Zn|Cu asymmetric cell was assembled with Zn foil (bare Zn or Zn@COF-S-F) as the anode, Cu foil as the cathode and 2 M ZnSO_4_ solution as electrolyte. Constant current charge–discharge cycle tests were carried out on the multi-channel cell test system (CT2001A, Wuhan Land). Galvanostatic testing of symmetric cells based on bare Zn or Zn@COF-S-F started after 4 h of assembly. The electrochemical workstation (Shanghai Chi 660E Chenhua) received the linear polarization curves, chronoamperogram (CA), cyclic voltammetry (CV), and electrochemical impedance spectroscopy (EIS). In 2 M ZnSO_4_ solution, the corrosion curve was carried out at a scanning rate of 10 mV s^−1^. EIS tests were performed in the frequency range of 10^5^ to 10^−2^ Hz.

### DFT Computations

The adsorption/desolvation energy was calculated using Gaussian 16 packages. Models for all computational molecules were optimized by PBEPBE functional with a 3–21G basis set. Besides, the Zn diffusion path through the COF-S-F coating was performed using the CP2K package. The electronic structure approach was adopted under the framework of mixed Gaussian and plane wave methods (GPW and GAPW). PBE functional was selected as exchange−correlation functional. In addition, Goedecker-Teter-Hutter (GTH) pseudopotentials and DZVP-MOLOPT-SR-GTH basis sets were used to describe the system. A plane-wave energy cutoff of 500 Ry and a relative cutoff of 45 Ry were adopted. The energy of SCF was set to 5.0 × 10^−6^ Ha.

### Multi-physical Simulation

The multi-physical field simulation is built and solved on COMSOL Multiphysics 6.0 software. Based on Gaussian random and uniform random distribution functions in COMSOL, we constructed a Zn metal anode surface with certain surface roughness and achieved subsequent solutions. The average roughness of the Zn metal anode surface is 14.841 µm and the size is 100 × 100 µm^2^. The thickness of the COF layer is 10 µm. The model is built by using ultrafine grid division, and the maximum grid size is 0.015 µm. Based on the classic Butler-Volmer equation, Faraday's laws of electrolysis, and the Nernst-Einstein relationship that takes into account the effect of the electric field, with the help of deformed mesh function in COMSOL, we realized a three-dimensional, dynamic solution to the Zn ion deposition process. The solution process is based on the MUMPS solver and does not consider the occurrence of any side reactions, that is, the electrodeposition efficiency is 100%.

## Results and Discussion

### Preparation Process and Action Mechanism of COF-S-F

**Fig. 1:**
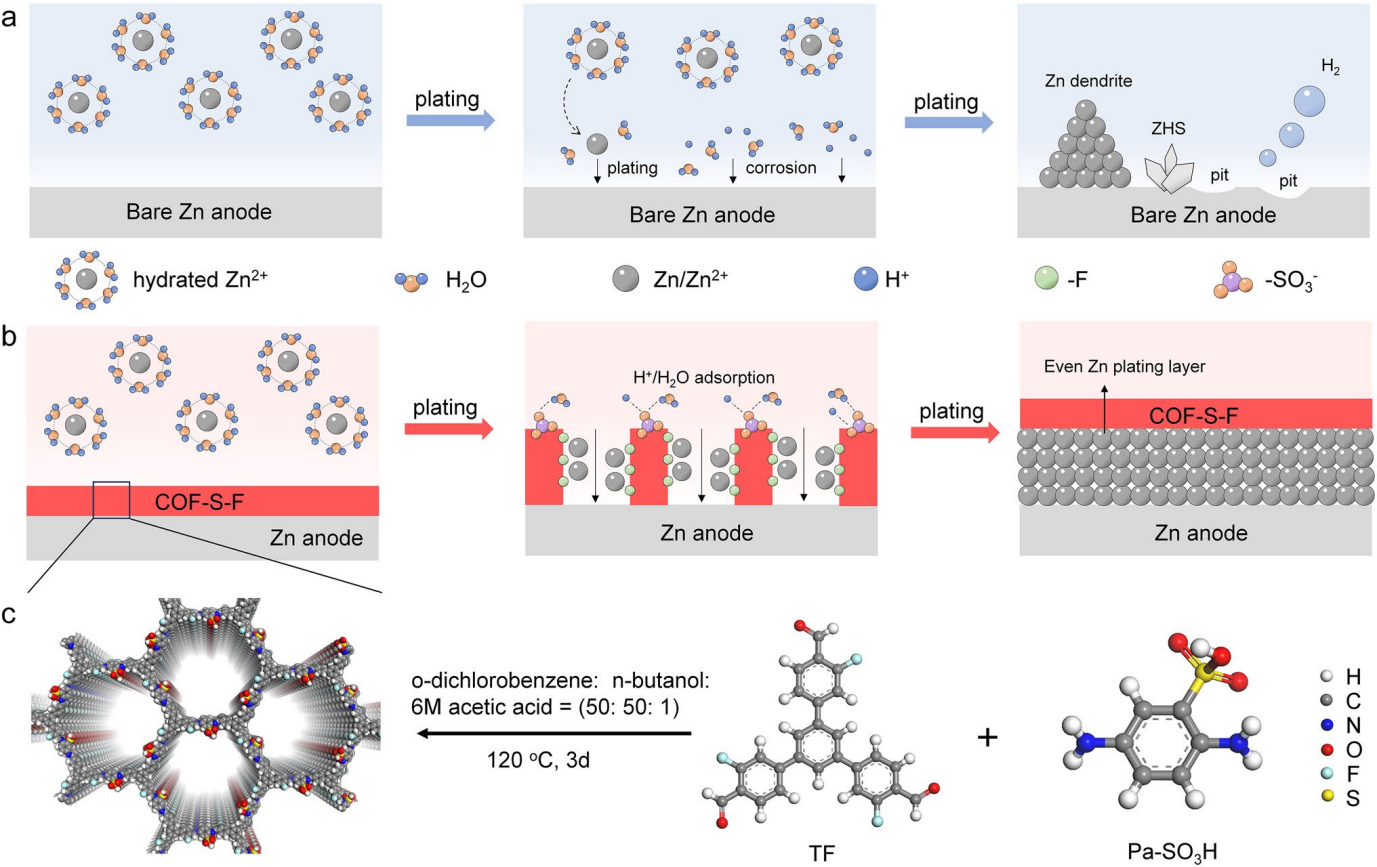
Preparation process and action mechanism of COF-S-F. Schematic comparison of Zn deposition process on **a** bare Zn and **b** Zn@COF-S-F. **c** Schematic diagram of the synthesis of COF-S-F

As shown in Fig. 1a, HER happens spontaneously when activated H_2_O molecules reach the surface of Zn, leading to the accumulation of by-products of Zn hexahydrate ([Zn(H_2_O)_6_]^2+^) with sulfate. Meanwhile, uneven interface morphology appears due to the electrodeposition of Zn metal in dendritic type. Hence, unpretreated Zn is very susceptible to corrosion, HER, morphologically unstable interface for the growth of dendrites, and the like issues [31, 32]. Taking the synergetic impacts of the structure and chemical component of COF-S-F, it can alter the structure of the double electric layer on the Zn anode surface, endowing Zn metal anode with outstanding structural stability during charging/discharging process (Fig. 1b, c). Specifically, the hydrophilic -SO_3_H group inside COF-S-F can immobilize the active H_2_O molecules and facilitate the desolvation process of hydrated Zn ions. Meanwhile, the highly electronegative and ionized -SO_3_^−^ group can capture H^+^ to further prevent its contact with Zn. The hydrophobic -F group yields a strong repulsion to H_2_O molecules, which improves Zn anode corrosion resistance. Structurally, the 3D porous structure of COF-S-F also guides the uniform transport of Zn ions and thus guarantees uniform electrodeposition morphology. Ultimately, outstanding-performance Zn anode with dendrite-free, corrosion-free is achieved by building artificial SEI on Zn metal with COF-S-F.

### Characterizations of Zn@COF-S-F

**Fig. 2:**
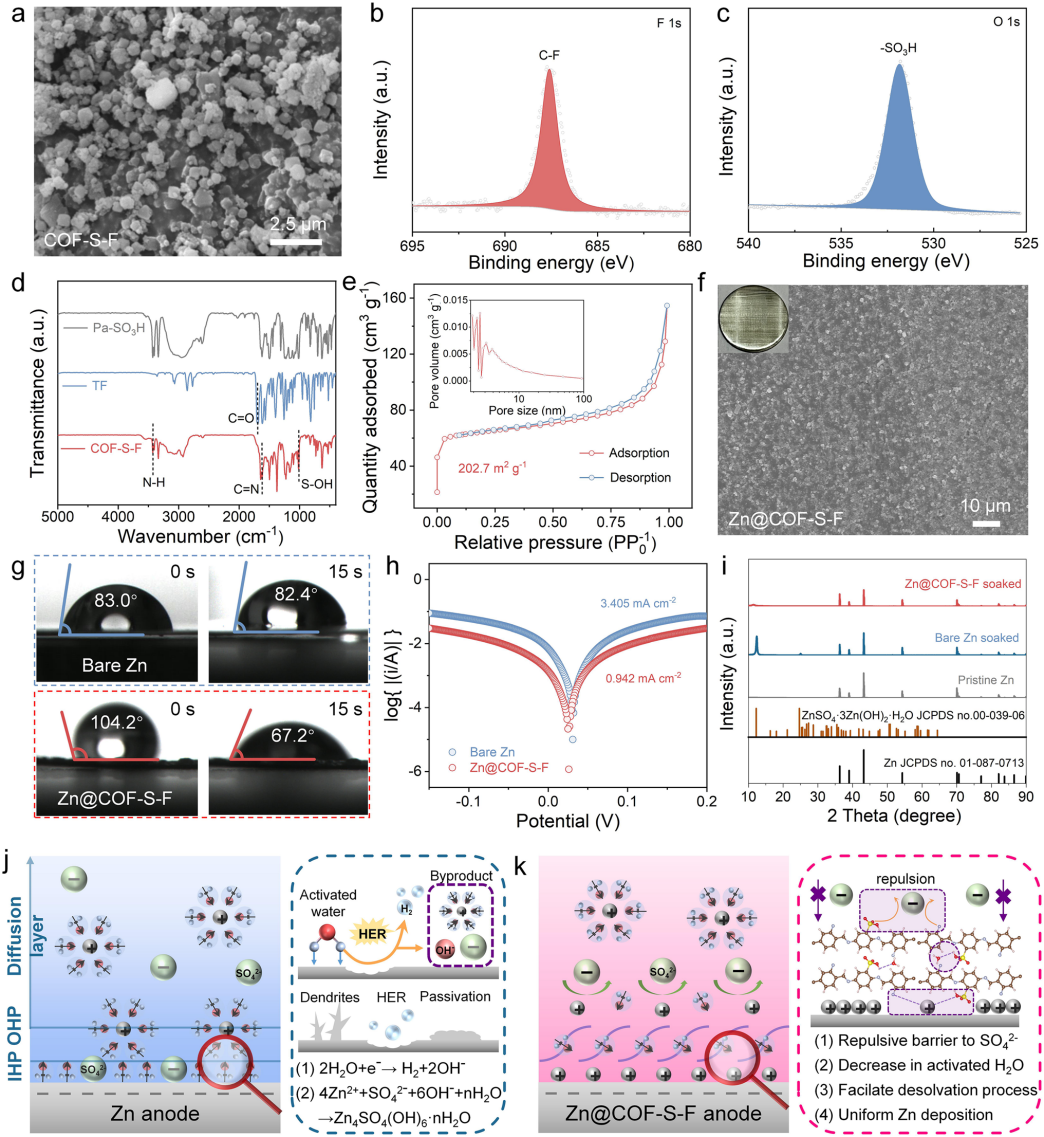
Characterizations of Zn@COF-S-F. **a** SEM image of as-prepared COF-S-F powder. High-resolution XPS **b** F 1*s* and **c** O 1*s* spectra of Zn@COF-S-F. **d** FT-IR spectrum of COF-S-F. **e** N_2_ adsorption/desorption isotherm and pore size distribution of COF-S-F. **f** SEM image and digital photo of Zn@COF-S-F. **g** Contact angle of H_2_O against bare Zn and Zn@COF-S-F after 0 s, 15 s. **h** linear polarization curves of bare Zn and Zn@COF-S-F symmetric cells. **i** XRD patterns of bare Zn and Zn@COF-S-F soaked in ZnSO_4_ electrolyte for three days. **j** Schematic diagram of the interfacial side reactions at bare Zn anode. **k** Mechanism of interfacial modification of Zn@COF-S-F anode

As shown in Fig. 2a, the COF-S-F powder presents uniform and loose, which is expected to facilitate the construction of flat coated Zn anode. Meanwhile, the elemental mapping shows that the COF-S-F possesses ample F and S elements (Fig. S1). The results of transmission electron microscopy (TEM) demonstrate that the COF-S-F powder exhibits a uniform pore structure (Fig. S2). The distinct (110) plane peak in the XRD pattern implies the crystalline structure of COF-S-F powder (Fig. S3) [33]. As shown in Figs. 2b, c and S4a, the appearance of the peaks located at 685.5, 532, and 168 eV in the high-resolution XPS spectra of F 1*s*, O 1*s*, S 2*p* proves the present of -SO_3_H and -F groups in as-prepared COF-S-F powder [34, 35]. Furthermore, the obvious peaks in XPS spectra of C 1*s* and N 1*s* confirm the benzene ring structure and imine bonds, respectively (Fig. S4b, c) [36, 37]. An obvious peak at 1015 cm^−1^ in Fourier transform infrared spectroscopy (FT-IR) of as-prepared COF-S-F powder can be identified as the stretching vibration of the S-OH bond, demonstrating the existence of -SO_3_H group in COF-S-F (Fig. 2d) [38]. Notably, the absorption peak at 1615 cm^−1^ is the C = N imide stretching vibration peak formed after the reaction [39], indicating that the formyl group in 1, 3, 5-tris(3-fluoro-4-formylphenyl) benzene (TF) undergoes a condensation reaction with the amino group in TpPa-SO_3_H. As shown in Fig. 2e, the hysteresis-free reversible type IV isotherm shows the distinct pore structure and large specific surface area (202.7 m^2^ g^−1^) of COF-S-F powder, providing more transport channels to facilitate the homogeneous and directional transport of Zn ions.

Following, the COF-S-F slurry is dropped on bare Zn metal surface to prepare Zn@COF-S-F, where COF-S-F is uniformly distributed in the spherical shape (Fig. 2f). As shown in Fig. 2g, contact angles of H_2_O against Zn@COF-S-F is 104.2° at 0 s and is sharply declined to 67.2° at 15 s with the continuous penetration of H_2_O, which can be explained as the result that impact of hydrophilic -SO_3_H group gradually excesses the hydrophobic effect of the -F group inside COF-S-F [40]. The COF-S-F coating improves the wettability of the Zn metal anode, and the increase in hydrophilicity reduces the interfacial free energy between the Zn metal and the electrolyte, resulting in a lower charge transfer resistance during Zn deposition/dissolution, which in turn improves the long-cycle performance of the cells. However, contact angles against bare Zn are higher as 83.0° and 82.4° at 0 and 15 s [31]. Consequently, owing to the existence of high-electronegative ionized -SO_3_^−^ group and hydrophobic -F group, the corrosion current density of Zn@COF-S-F symmetric cell is decreased from 3.405 mA cm^−2^ of bare Zn symmetric cell to 0.942 mA cm^−2^ (Fig. 2h) [41]. Although PVDF also contains F element, the corrosion current density of Zn@PVDF symmetric cell is 1.968 mA cm^−2^ (Fig. S5), indicating that it is mainly F element in COF that plays the role. Furthermore, the bare Zn and Zn@COF-S-F are immersed in the electrolyte for three days to investigate side reaction with aqueous electrolyte. Compared with the existence of intense peaks belonging to ZnSO_4_·3Zn(OH)_2_·5H_2_O in XRD pattens of bare Zn that soaked in electrolyte, weak peak intensity of by-products in that of Zn@COF-S-F confirms that severe corrosion against aqueous electrolyte can be prevented significantly with COF-S-F coating (Fig. 2i). Less existence of by-products on the surface of Zn@COF-S-F after immersion is also confirmed by SEM (Fig. S6). Therefore, the -SO_3_H and -F groups in COF-S-F can effectively inhibit the notorious side reaction against aqueous electrolyte, which is regarded as the guarantee for building high-performance Zn anode with superior interfacial stability [42]. As shown in Fig. 2j, when activated H_2_O molecules reach the surface of Zn, they trigger the HER and combine with sulfate to produce by-products [43]. Conversely, the COF-S-F coating alters the structure of the double electric layer on the Zn anode surface, allowing the Zn anode to remain more stable during charging and discharging (Fig. 2k). The highly electronegative sulfonic acid groups and fluorine atoms can immobilize the active water molecules in the electrolyte and facilitate the desolvation process of hydrated Zn ions. On the other hand, the migration of sulfate from the electrolyte to the Zn anode is repelled due to electrostatic repulsion, and the large amount of H^+^ ionized by the sulfonic acid group neutralizes the OH^−^ obtained from the ionized water, thus inhibiting the formation of by-products.

### Theoretical Calculations and Simulations of Zn@COF-S-F

**Fig. 3:**
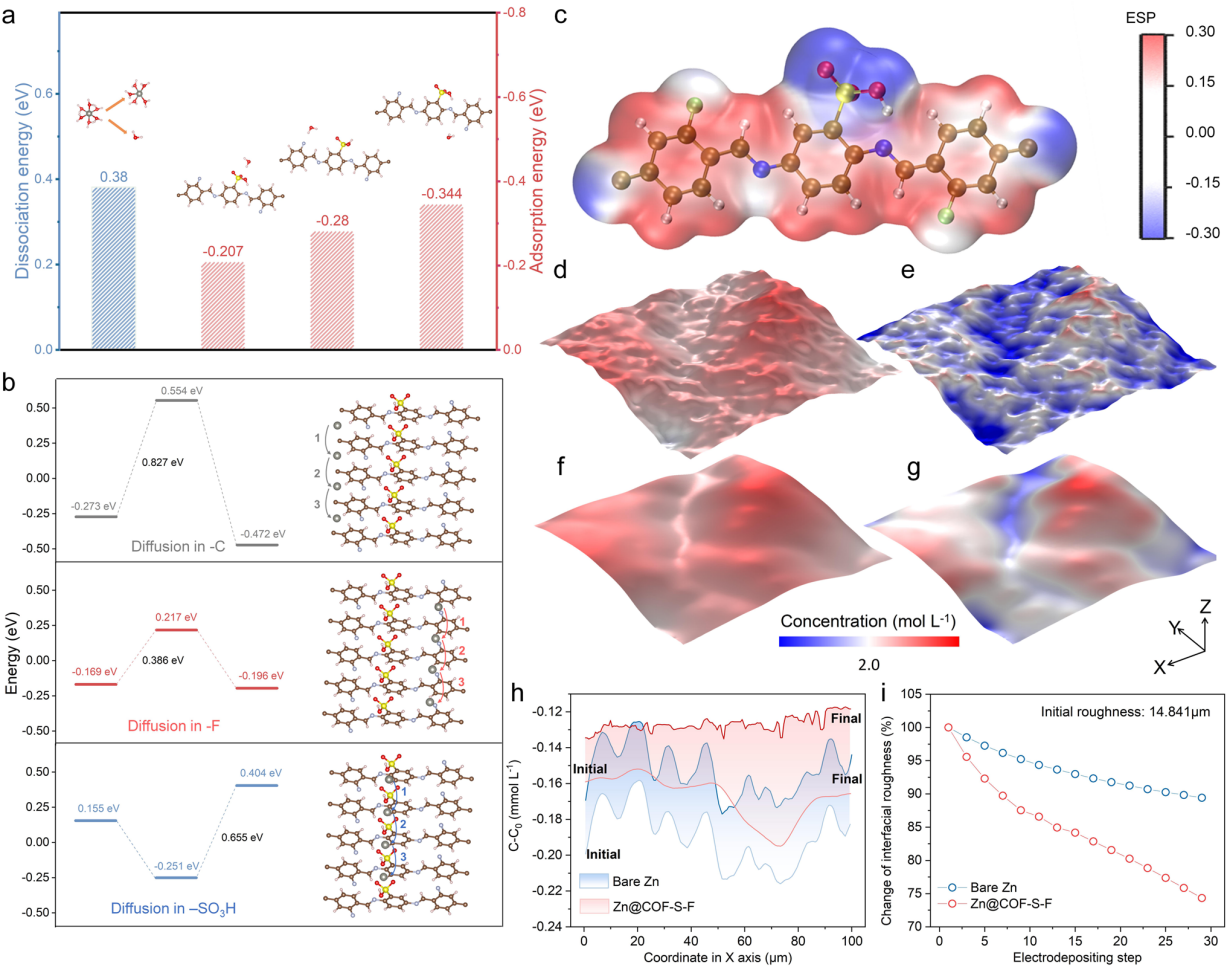
Desolvation and confinement of COF-S-F. **a** Comparison of the dissociation energy of [Zn(H_2_O)_6_]^2+^ with the adsorption energy between H_2_O and different groups of COF-S-F. **b** Migration pathways of Zn ion and the diffusion energy barrier of Zn ion in COF-S-F. **c** Electrostatic potential of COF-S-F molecules. Distribution of Zn-ion concentration on **d** Zn@COF-S-F surface and **e** bare Zn metal at initial stage with random-generated surface structure. Final morphology with distribution of concentration field of **f** Zn@COF-S-F and **g** bare Zn. **h** Variation of concentration along X-axis at Y = 50 µm in Fig. 3d-g. **i** Change of interfacial roughness along with the electrodeposition, of which initial roughness is 14.841 µm

The density functional theory (DFT) is conducted to reveal the impact of COF-S-F on the desolvation process of hydrated Zn ions ([Zn(H_2_O)_6_]^2+^) and the diffusion of Zn ions through COF-S-F. As shown in Fig. 3a, the dissociation energy for [Zn(H_2_O)_6_]^2+^ to lose one H_2_O molecule is 0.38 eV, while the adsorption energy of H_2_O molecule to different functional groups, -F, -C, -SO_3_H in COF-S-F to water is − 0.207, − 0.28 and − 0.344 eV, respectively, indicating that the solvated water molecules around the [Zn(H_2_O)_6_]^2+^ will be attracted by the -SO_3_H groups. Furthermore, the migration behavior of Zn ion in COF-S-F is further studied. Figure 3b shows the diffusion path of Zn ions at three different functional groups in COF-S-F. The energy barriers of Zn ion across -F group in COF-S-F is the lowest (0.386 eV) among that across other groups (0.827 eV for -C and 0.655 eV for -SO_3_H), due to the negative electrostatic potentials (blue region) of -F and -SO_3_H with strong electrostatic interaction with Zn ions (Fig. 3b, c). Therefore, the desolvation process of [Zn(H_2_O)_6_]^2+^ is promoted and the migration of Zn ions across the COF-S-F layer is accelerated [44].

In addition, the multi-physical field simulation, a powerful tool to reveal concentration field evolution along the electrochemical process, is conducted based on the Butler-Volmer equation [45]:

1 $$i= i_{0} \left[ {{\text{exp}}\left( {\frac{{\alpha F}}{{RT}}\eta } \right) - \frac{{c_{{Zn^{{2 + }} }} }}{{c_{0} }}{\text{exp}}\left( {\frac{{(1 - \alpha )F}}{{RT}}\eta } \right)} \right]$$

with current density ($i$), exchange current density (${i}_{0}$), anodic reaction constant ($\alpha$), Faradic constant ($F$), ideal gas constant ($R$), Kelvin temperature ($T$), overpotential ($\eta$), the concentration of Zn ion (${c}_{{Zn}^{2+}}$, ${c}_{0}$) to describe the electrodeposition process of Zn metal under the impact of COF-S-F artificial SEI (see details in Multi-physical simulation) [46, 47]. First, the initial surface with a roughness of 14.841 µm is generated by the random function and the size of the as-generated surface is 100 µm × 100 µm. And the simulated geometric model is shown in Fig. S7. As shown in Fig. 3d, e, the concentration of Zn ions is distributed uniformly under the cover of COF-S-F artificial SEI, compared with the obvious existence of concentration “hot spots” on the protrusion. It is notable that COF-S-F artificial SEI with -F group on confined channels can uniform the flux of Zn ions onto the surface and realize the uniform concentration field. Furthermore, along with the electrodeposition of Zn, the concentration of Zn ions on Zn@COF-S-F is continually even while that on bare Zn metal is always uneven. Moreover, the electrodeposited surface Zn@COF-S-F is flatter and smoother than that of bare Zn (Fig. 3f, g and Videos S1, S2). To be more specific, the variation of concentration of Zn ion on the Zn metal surface is sliced at Y = 50 µm in Fig. 3f, g. As shown in Fig. 3h, both of concentration of Zn ions and the corresponding uniformity on Zn@COF-S-F is reinforced, while bare Zn metal witnesses continuously uneven concentration of Zn ion from the initial to the final stage. Moreover, the change of interfacial roughness is defined as the ratio between the roughness of the evolving surface to the initial roughness of 14.841 µm, which can be conducted to evaluate the electrodeposition uniformity of Zn metal even with a rough initial surface. It can be found that the final roughness of Zn@COF-S-F is 11.031 µm while that of bare Zn is 13.272 µm, and the smoothing rate of Zn@COF-S-F is accelerated compared to that of bare Zn metal (Fig. 3i). Thus, taking the synergetic impact of advantage of -SO_3_H groups on the desolvation process of [Zn(H_2_O)_6_]^2+^ and confined channel with -F group on uniformization of flux of Zn ions, the corrosion-free and dendrite-free Zn metal with satisfactory electrochemical performance can be predicted [48].

### Electrochemical Performance of Zn@COF-S-F Symmetric Cells

**Fig. 4:**
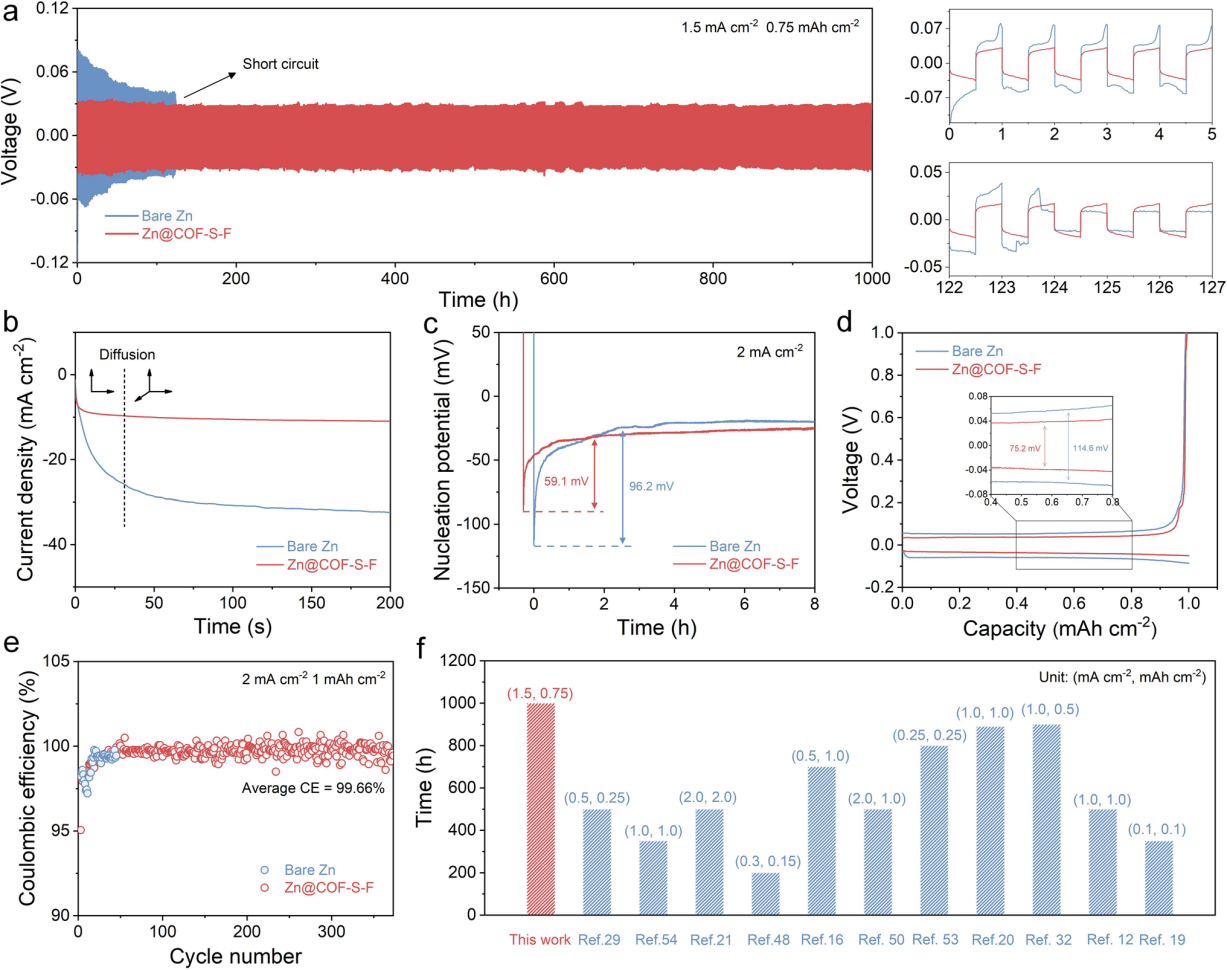
Enhancements in stability and reversibility by Zn@COF-S-F. **a** Galvanostatic charge/discharge cycling voltage profiles of bare Zn and Zn@COF-S-F symmetric cells. **b** Chronoamperometry profiles of bare Zn and Zn@COF-S-F symmetric cells at the overpotential of − 200 mV. **c** Nucleation overpotentials of bare Zn|Cu and Zn@COF-S-F|Cu asymmetric cells. **d** Plating/stripping profiles of bare Zn|Cu and Zn@COF-S-F|Cu asymmetric cells at a current density of 2 mA cm^−2^ with a capacity of 1 mAh cm^−2^. **e** Coulombic efficiency of Zn|Cu and Zn@COF-S-F|Cu asymmetric cells. **f** Comparison of cyclic reversibility with other previous reports

As shown in Fig. S8, cyclic voltammetry (CV) curves of Zn@COF-S-F|Ti asymmetric cell exhibit higher peak current densities than that of bare Zn|Ti asymmetric cell, demonstrating that the electrochemical kinetics of Zn plating/stripping is fostered with COF-S-F coating. Following, the long-term plating/stripping performances of bare Zn and Zn@COF-S-F symmetric cells are implemented to verify the effectiveness of COF-S-F artificial SEI film. At the gentle current density of 0.3 mA cm^−2^, the bare Zn symmetric cell suffers from serious polarization at the beginning, and it shorts less than 200 h eventually. And Zn@COF-S-F symmetric cell shows prolongated cycling stability up to 500 h with smaller voltage hysteresis compared with bare Zn symmetric cell (62.1 *vs*. 78.6 mV) (Fig. S9). When higher current density of 1.5 mA cm^−2^ is applied, the Zn@COF-S-F symmetric cell maintains the cycling stability for 1,000 h with the lower voltage hysteresis of 50.5 mV, while the bare Zn symmetric cell is short-circuited after 123 h (Figs. 4a and S10). Moreover, the rate performances of symmetric cells at stepped current densities are compared to evaluate the effect of COF-S-F on the electrochemical kinetics of Zn plating/stripping [49]. As the current density increases from 0.25 to 4.0 mA cm^−2^, the corresponding polarization voltages display a minor increase from 41 to 57 mV for Zn@COF-S-F symmetric cells, which are lower than those of bare Zn and Zn@PVDF symmetric cells (Fig. S11). Then, chronoamperogram (CA) is conducted to investigate the changes of Zn nucleation and during electrodeposition. As shown in Figs. 4b and S12, when the constant overpotential is applied as − 200 mV, the current of bare Zn and Zn@PVDF symmetric cells changes significantly and declines continuously for 200 s, while that of Zn@COF-S-F symmetric cells holds steady after only 5 s, indicating that long two-dimensional diffusion process of Zn ions on bare Zn surface with disordered nucleation has been regulated as short three-dimensional diffusion process with uniform nucleation [50]. Moreover, the overpotential for nucleation of Zn@COF-S-F|Cu asymmetric cell (59.1 mV) is smaller than Zn@PVDF|Cu (87.6 mV) and bare Zn|Cu (96.2 mV) asymmetric cell (Figs. 4c and S13), illustrating the facilitated nucleation process during the electro-crystallization of Zn metal. Thus, the uniform and flat electrodeposited Zn@COF-S-F can be expected reasonably from the classical nucleation theory [45]. On the other hand, the polarization of Zn@COF-S-F|Cu asymmetric cell (75.2 mV) is smaller than that of bare Zn|Cu (114.6 mV) asymmetric cells (Fig. 4d). As expected, Zn@COF-S-F|Cu asymmetric cell shows higher average Coulombic efficiency (CE) of 99.66% at current density of 2.0 mA cm^−2^ and maintains steady for more than 350 cycles, which is more outstanding than the 50-cycle-life bare Zn|Cu asymmetric cell (Fig. 4e). Hence, the persistence and reversibility of Zn plating/stripping process is significantly reinforced due to the design of COF-S-F film [51, 52]. To further investigate the impact of COF-S-F on the inhibition of dendrite growth and generation of by-products, SEM and XRD tests are carried out on different Zn anodes after 500 h of cycling. Compared with uneven bare Zn surface with massive by-products, Zn@COF-S-F exhibits a flatter and denser surface, which are further confirmed by XRD patterns (Figs. S14 and S15). Promisingly, although other studies in this area demonstrate good performances, the designs and performances of this work are more profound, effective and outstanding (Fig. 4f and Table S1) [16, 20, 21, 32, 33, 53].

### Electrochemical Performance of Zn@COF-S-F|MnO_2_ Full Cells

**Fig. 5:**
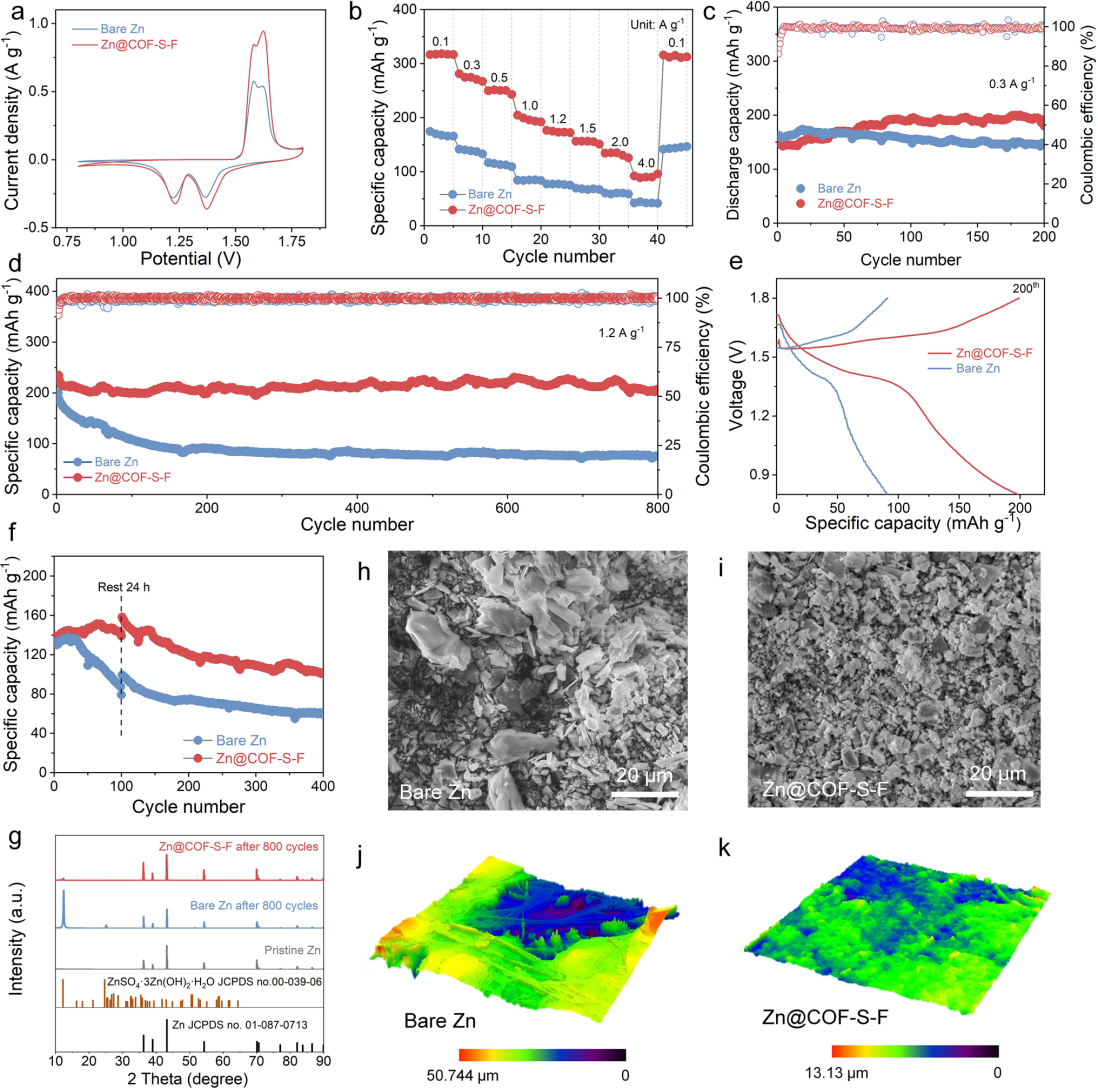
Full cells enhanced electrochemical performance with COF-S-F. **a** CV curves of bare Zn|MnO_2_ and Zn@COF-S-F|MnO_2_ full cells. **b** Rate performances of bare Zn|MnO_2_ and Zn@COF-S-F|MnO_2_ full cells. Cycling performances of bare Zn|MnO_2_ and Zn@COF-S-F|MnO_2_ full cells at current density of 0.3 **c** and 1.2 **d** A g^−1^. **e** Galvanostatic charge/discharge curves of bare Zn|MnO_2_ and Zn@COF-S-F|MnO_2_ full cells at 200th cycle. **f** Cycling performances after resting for 24 h of bare Zn|MnO_2_ and Zn@COF-S-F|MnO_2_ full cells at current density of 0.5 A g^−1^. SEM images of **h** bare Zn and **i** Zn@COF-S-F anode after 800 cycles. **g** XRD patterns of bare Zn and Zn@COF-S-F anode after 800 cycles. 3D LCSM images of **j** bare Zn and **k** Zn@COF-S-F anode after 800 cycles

Eventually, the full cells with α-MnO_2_ cathode are assembled to assess the practical role of Zn@COF-S-F anode (Fig. S16). As shown in Fig. 5a, the CV curves have the consistent peak shape with same positions, indicating that the introduction of COF-S-F artificial SEI does not alter the related electrochemical process [53, 54]. Besides, the redox peak current densities of the Zn@COF-S-F|MnO_2_ full cell are 0.94/0.32 A and 0.85/0.36 A g^−1^, respectively, which are both higher than those of the bare Zn|MnO_2_ cell (0.58/0.28 and 0.54/0.27 A g^−1^). Hence, the Zn@COF-S-F|MnO_2_ full cell delivers high electrochemical activity and lower polarization. Zn@COF-S-F|MnO_2_ full cell at stepped current densities ranging from 0.1 to 4.0 A g^−1^ can deliver higher capacities, which are higher than those of bare Zn|MnO_2_ full cell. Then the capacity of Zn@COF-S-F|MnO_2_ full cell can be restored back to 315.2 mAh g^−1^ when the current density is back to 0.1 A g^−1^ (Figs. 5b and S17). Compared with the continuous capacity fading of bare Zn|MnO_2_ full cell at the current density of 0.3 A g^−1^, Zn@COF-S-F|MnO_2_ full cell still delivers the high specific capacity up to 190.1 mAh g^−1^ after 200 cycles with stable CE and charge/discharge voltage platform (Figs. 5c and S18). Moreover, when the current density increases to 1.2 A g^−1^, the initial discharge specific capacity of Zn@COF-S-F|MnO_2_ full cell (235.3 mAh g^−1^) is higher than that of the bare Zn|MnO_2_ full cell (199.9 mAh g^−1^). After 800 cycles, Zn@COF-S-F|MnO_2_ cell embraces an outstanding capacity retention rate of 87.9% with a more stable CE and charge–discharge curve, while that of bare Zn|MnO_2_ cell is only 37.9% (Fig. 5d, e). Furthermore, the protection impact of COF-S-F layer is investigated by the self-discharge test. As shown in Fig. 5f, Zn@COF-S-F|MnO_2_ cell maintains a higher discharge capacity after shelving for 24 h at 100 th cycle, and it still can deliver discharge specific capacity of 100.7 mAh g^−1^ after 400 cycles, which is much improved compared with bare Zn|MnO_2_ cell (59.7 mAh g^−1^). Moreover, the Zn@COF-S-F|MnO_2_ cell is more stable and less polarized at the 100th charge/discharge platform (Fig. S19). Therefore, the cyclic performance of the full cell is significantly improved with excellent self-discharge resistance, which is attributed to the introduction of COF-S-F film with regulated desolvation process of hydrated Zn ions and faster diffusion rate for Zn ions to uniform the electrodeposition of Zn metal.

Following, the anodes are disassembled from the full cells after 800 cycles to further confirm the protection of COF-S-F film on the Zn metal anode. As shown in Fig. 5g, the appearance of intense characteristic peaks that are identified as ZnSO_4_·3Zn(OH)_2_·5H_2_O in the XRD pattern of bare Zn anode demonstrates the occurrence of severe side reactions. Whereas, the related characteristic peak of by-products in the XRD pattern of Zn@COF-S-F is obviously inferior, proving that the introduction of COF-S-F film can effectively weaken the side reaction and thus lessen the accumulation of by-products. Because of the certain desolvation effect of COF-S-F, the contact between active H_2_O molecules and metallic Zn is blocked, thus inhibiting the production of ZnSO_4_·3Zn(OH)_2_·0.5H_2_O, and the corresponding diffraction peak is not obvious. Furthermore, SEM and laser scanning confocal microscopy (LSCM) are conducted to investigate the morphology of Zn metal that protected by COF-S-F film. From the SEM images in Figs. 5h, i and S20, a large number of Zn dendrites appear on the bare Zn anode, while the surface of Zn@COF-S-F anode is relatively flat with negligible impurities. Furthermore, compared with the giant height difference of bare Zn anode of 50.744 µm, the surface of the Zn@COF-S-F anode is relatively uniform with low roughness of 13.13 µm (Fig. 5j, k). Hence, the introduction of COF-S-F film can validly uniform the electrodeposition of Zn metal with dendrite-free. Taking these synergistic impacts of COF-S-F on weakening the side reaction and suppressing the growth of dendritic Zn, Zn@COF-S-F anode embraces superiority to extend the lifespan of Zn|MnO_2_ full cell.

## Conclusion

In this work, a fluorinated covalent organic framework (COF-S-F) with sulfonic acid group (-SO_3_H) is developed on the surface of Zn anode. The hydrophilic -SO_3_H group and the hydrophobic -F groups can facilitate the desolvation of the hydrated Zn ions, thereby inhibiting the side reactions with interfacial passivation. Meanwhile, the highly electronegative -F group in COF-S-F promotes the fast and uniform transport of Zn ions along the arrangement by electrostatic interactions, which contribute to the uniform electrodeposition process of Zn metal. Therefore, Zn@COF-S-F symmetric cell exhibits outstanding cycling stability against aqueous electrolyte, which can stably cycle for 1,000 cycles at the current density of 1.5 mA cm^−2^ with lower voltage hysteresis of 50.5 mV. In addition, Zn@COF-S-F|MnO_2_ full cell still delivers a high discharge specific capacity 206.8 mAh g^−1^ at the current density of 1.5 A g^−1^ after 800 cycles, embracing higher capacity retention of 87.9%. In summary, the synergetic effect of rapid desolvation and ion-confinement from COF-S-F realizes the dendrite-free and high-stable Zn metal anode, which effectively improves the electrochemical performance of AZIBs and thus faster practicalization.

## Supplementary Information

Below is the link to the electronic supplementary material.

Supplementary file1 (PDF 1431 KB)
