# Supplementary material for: Covalent Organic Framework with 3D Ordered Channel and Multi-Functional Groups Endows Zn Anode with Superior Stability
Source: Nanomicro Lett. 2024 Jan 4;16:76. doi: 10.1007/s40820-023-01278-0 (PMC10767043; doi:10.1007/s40820-023-01278-0)
Supplement: Supplementary file 1 — Supplementary file1 (PDF 1431 KB) [file 40820_2023_1278_MOESM1_ESM.pdf]

Supporting information for

Covalent Organic Framework with 3D Ordered Channel and Multi-Functional Groups Endows Zn Anode with Superior Stability

Bin Li[1,#], Pengchao Ruan[2,#], Xieyu Xu[3], Zhangxing He[1,*], Xinyan Zhu[1], Liang Pan[1], Ziyu Peng[1], Yangyang Liu[3,*], Peng Zhou[4], Bingan Lu[5], Lei Dai[1], and Jiang Zhou[2,*]

[1] School of Chemical Engineering, North China University of Science and Technology, Tangshan 063009, P. R. China

[2] School of Materials Science and Engineering, Central South University, Changsha 410083, P. R. China

[3] State Key Laboratory for Mechanical Behavior of Materials, Xi'an Jiaotong University, Xi'an 710049, P. R. China

[4] Hunan Provincial Key Defense Laboratory of High Temperature Wear-Resisting Materials and Preparation Technology, Hunan University of Science and Technology, Xiangtan 411201, P. R. China

[5] School of Physics and Electronics, Hunan University, Changsha, 410082, P. R. China

[#]Bin Li and Pengchao Ruan have contributed equally to this work.

[*]Corresponding authors. E-mail address: zxhe@ncst.edu.cn (Z. He); liuyy0510@hotmail.com (Y. Liu); zhou_jiang@csu.edu.cn (J. Zhou)

Supplementary Figures and Table

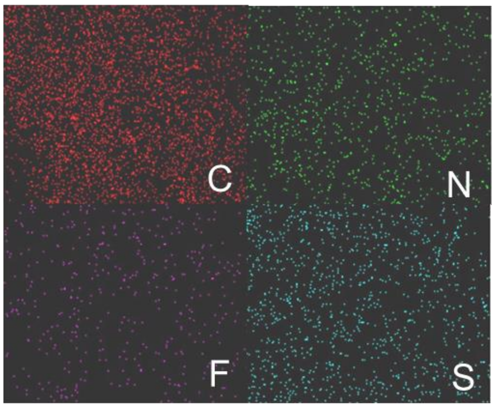

Fig. S1 Element mapping images of COF-S-F

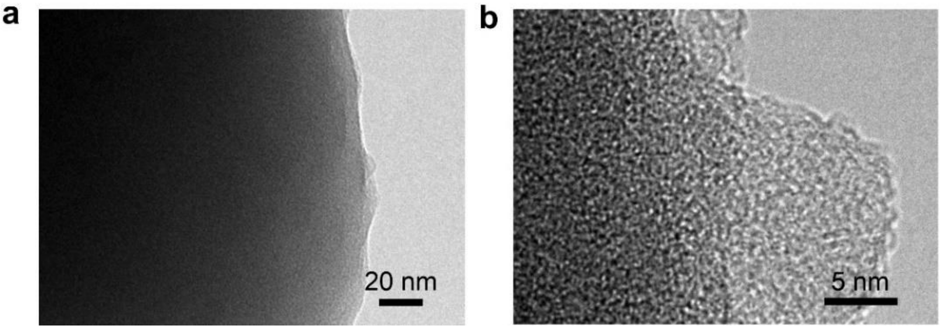

Fig. S2 TEM images of COF-S-F at (**a**) low magnification and (**b**) high magnification

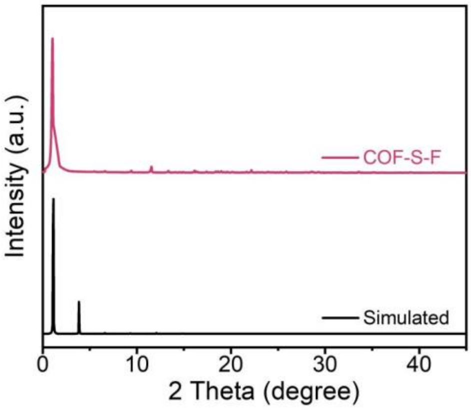

Fig. S3 XRD pattern of COF-S-F

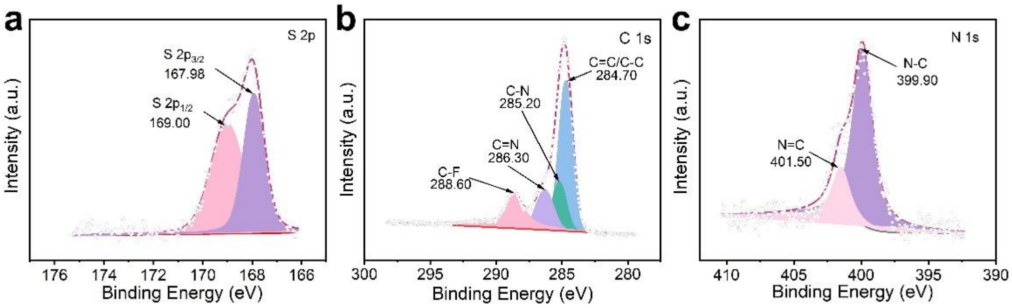

Fig. S4 High-resolution XPS spectra of COF-S-F for (**a**) S 2p, (**b**) C 1s and (**c**) N 1s

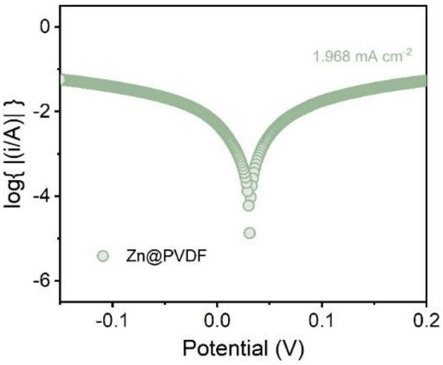

Fig. S5 linear polarization curve of Zn@PVDF symmetric cell

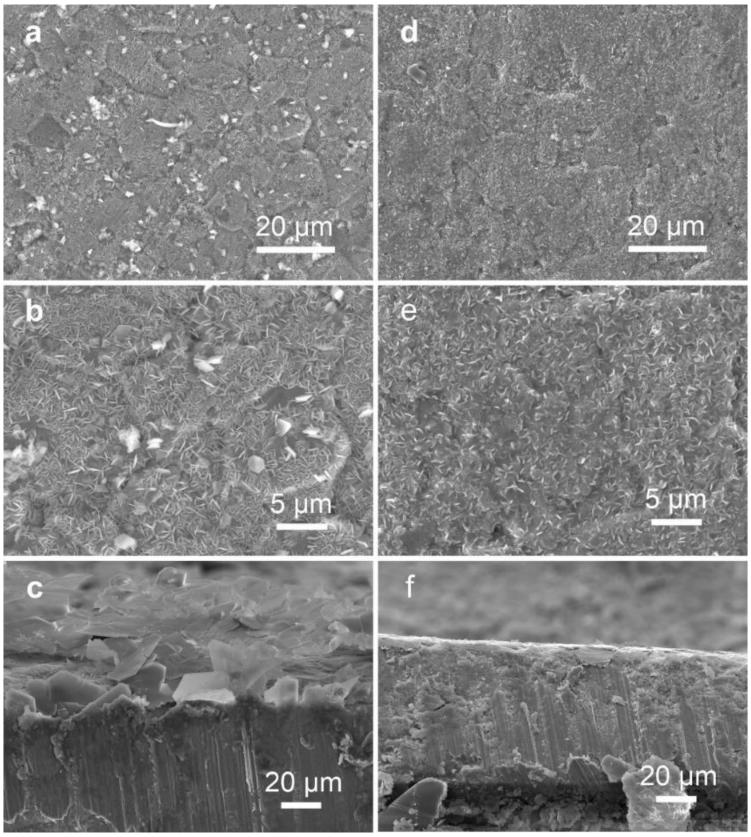

Fig. S6 SEM images of bare Zn (**a-c**) and Zn@COF-S-F (**d-f**) after 3-day-immersion in 2 M $ZnSO_4$ electrolyte

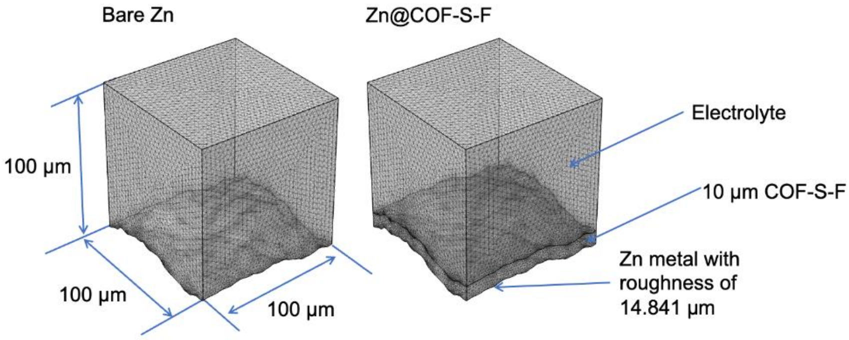

Fig. S7 The geometric model of the multi-physical simulation

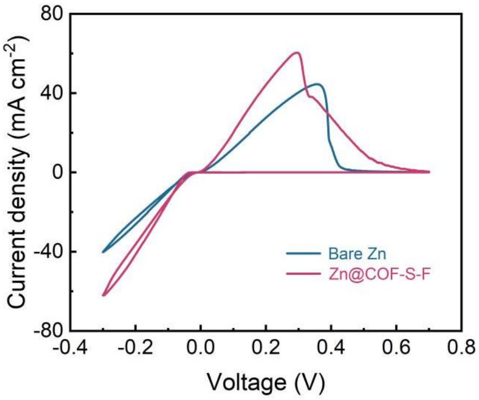

Fig. S8 CV curves of bare Zn|Ti and Zn@COF-S-F|Ti cells

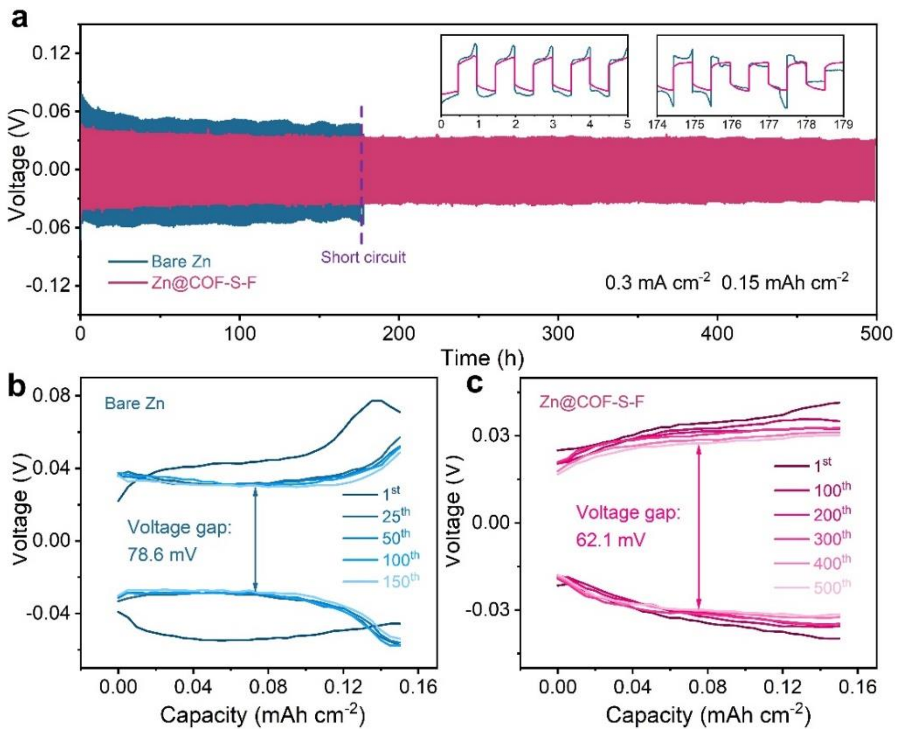

Fig. S9 (**a-c**) Galvanostatic charge/discharge cycling voltage profiles of bare Zn and Zn@COF-S-F symmetric cells at current density of 0.3 mA cm^{-2}

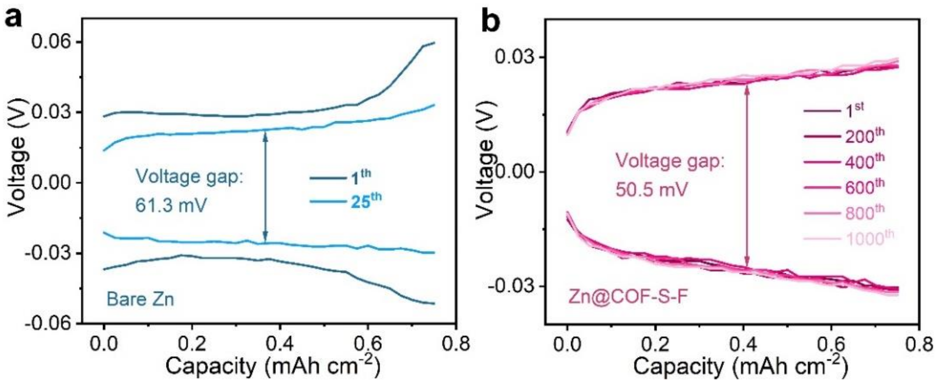

Fig. S10 Capacity voltage curves for bare Zn and Zn@COF-S-F symmetric cells at current density of 1.5 mA cm^{-2}

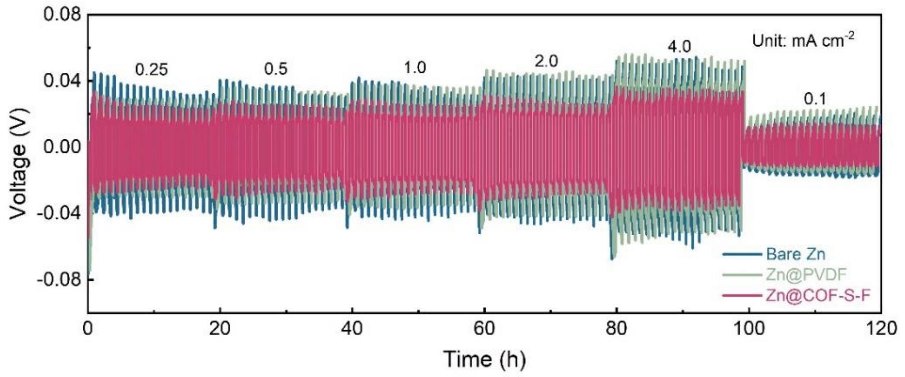

Fig. S11 Rate performance of bare Zn, Zn@COF-S-F and Zn@PVDF symmetrical cells

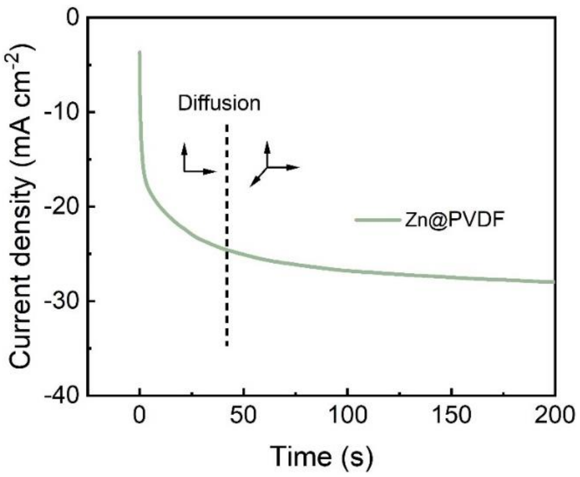

Fig. S12 Chronoamperometry profiles of Zn@PVDF symmetric cells at the overpotential of -200 mV

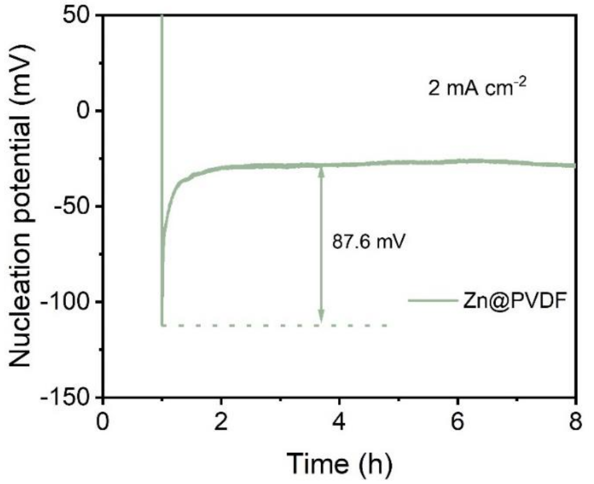

Fig. S13 Nucleation overpotentials of Zn@PVDF|Cu asymmetric cell

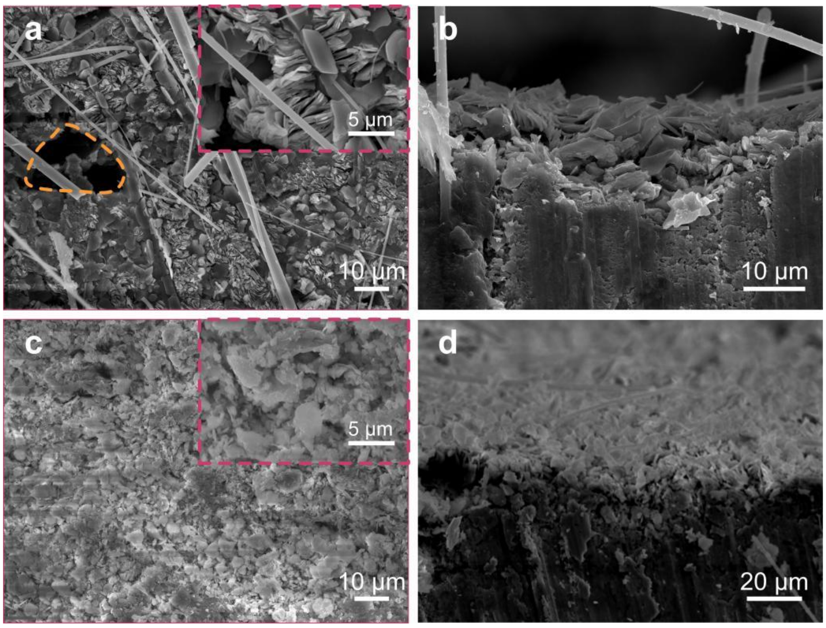

Fig. S14 SEM images of (**a, b**) bare Zn and (**c, d**) Zn@COF-S-F after 500 h of cycling

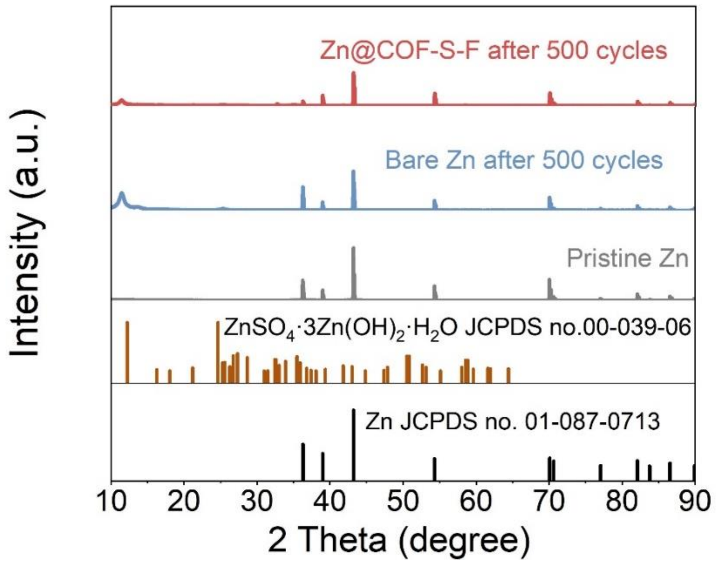

Fig. S15 XRD patterns of bare Zn and Zn@COF-S-F after 500 h of cycling

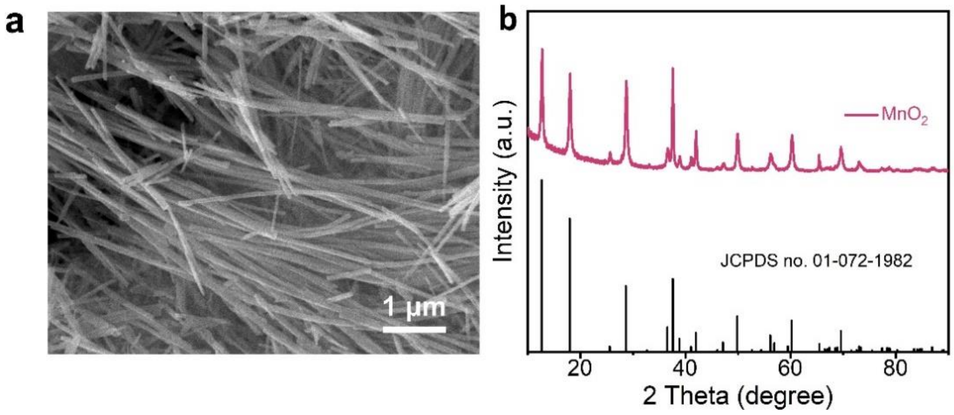

Fig. S16 (**a**) SEM image and (**b**) XRD pattern of MnO_2 cathode material

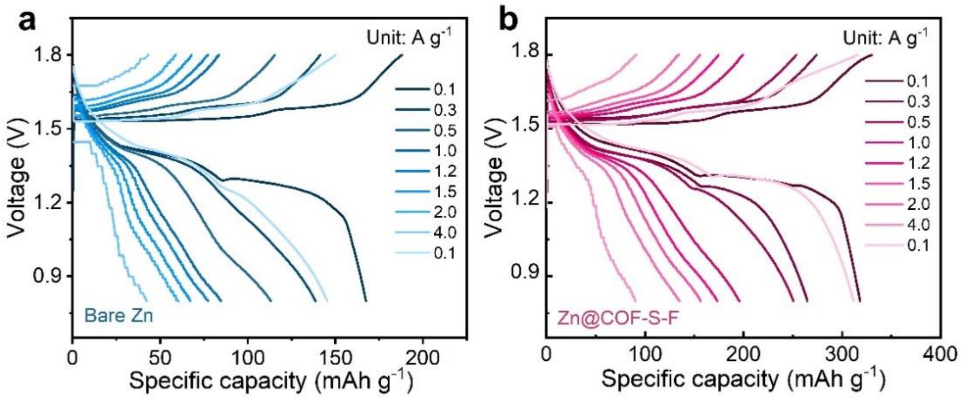

Fig. S17 Charge/discharge profiles of Zn|MnO_2 full cells with (**a**) bare Zn and (**b**) Zn@COF-S-F anodes at stepped current density ranging from 0.1 to 4.0 A g^{-1}

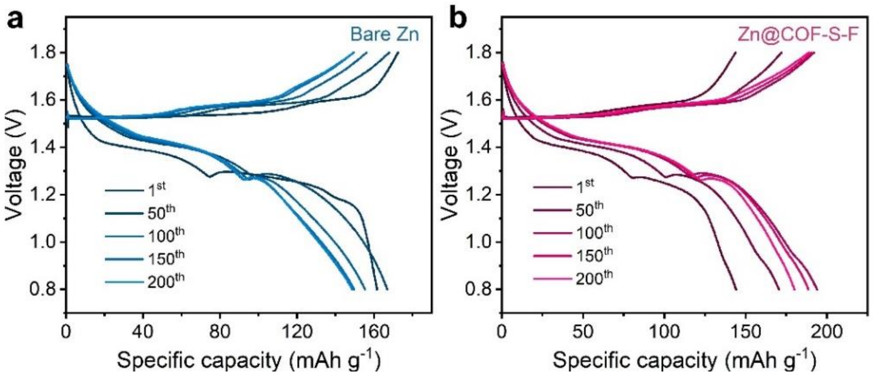

Fig. S18 Charge/discharge profiles of Zn|MnO_2 full cells with (**a**) bare Zn and (**b**) Zn@COF-S-F anode at current density of 0.3 A g^{-1} at different cycles

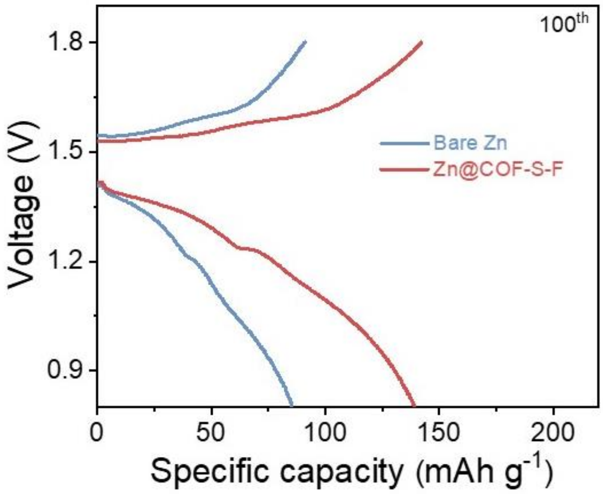

Fig. S19 Charge/discharge profiles of the rest test at 100th cycle at current density of 0.5 A g^{-1}

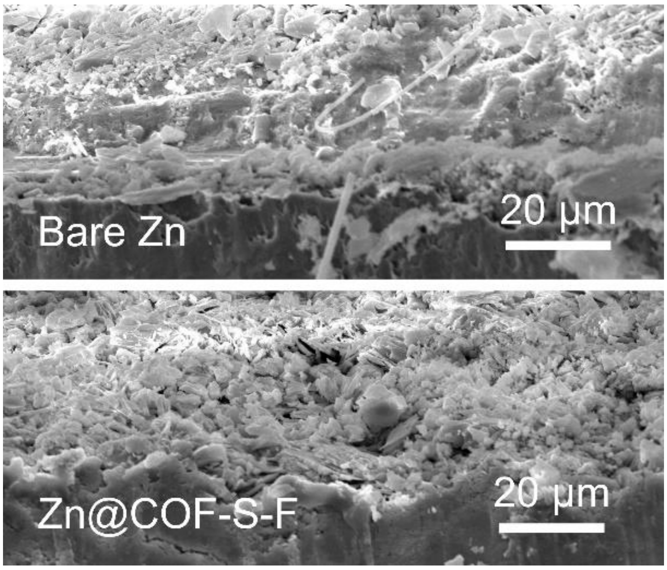

Fig. S20 Cross-sectional SEM images of the bare Zn and Zn@COF-S-F after 800 cycles at current density of 1.2 A g^{-1}

Table S1 Cycling performance comparison of this work and other previous reports

Work	Current density (mA cm^{-2})	Area specific capacity (mAh cm^{-2})	Cycling time (hour)
This work	1.5	0.75	1000
Ref.30	0.5	0.25	500
Ref.48	1.0	1.0	350
Ref.18	2.0	2.0	500
Ref.47	0.3	0.15	200
Ref.13	0.5	1.0	700
Ref.44	2.0	1.0	500
Ref.45	0.25	0.25	800
Ref.17	1.0	1.0	890
Ref.29	1.0	0.5	900
Ref.9	1.0	1.0	500
Ref.16	0.1	0.1	350
